# Supplementary material for: In silico exploration of phenolics as modulators of penicillin binding protein (PBP) 2× of Streptococcus pneumoniae
Source: Sci Rep. 2024 Apr 16;14:8788. doi: 10.1038/s41598-024-59489-3 (PMC11021432; doi:10.1038/s41598-024-59489-3)
Supplement: Supplementary file 1 — Supplementary Information. [file 41598_2024_59489_MOESM1_ESM.docx]

Table S1: Consensus phenolic pharmacophore spatial arrangements

| Pharmacophore class | x | y | z | Radius |
| --- | --- | --- | --- | --- |
| Aromatic | 115.78 | 65.05 | 80.39 | 1.10 |
| Hydrogen donor | 115.22 | 63.36 | 75.66 | 0.50 |
| Hydrogen acceptor | 119.96 | 59.19 | 75.03 | 0.50 |
| Hydrogen acceptor | 113.83 | 64.99 | 77.25 | 0.50 |
| Hydrophobic | 115.78 | 65.07 | 80.39 | 1.00 |


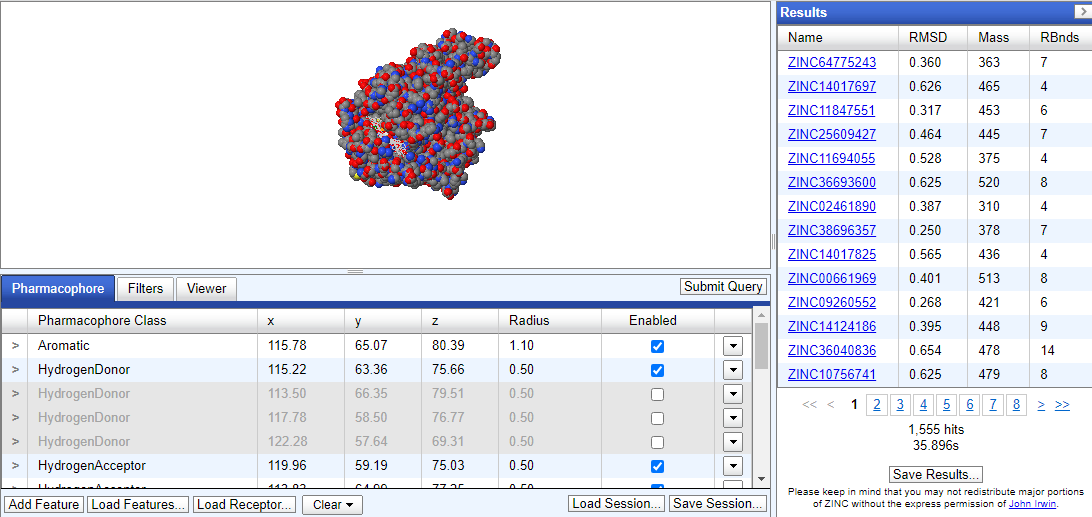


Figure S1: Observed 1555 hit phenolics

| Systems | Δ E_vdW_ | ΔE_elec_ | ΔG_gas_ | ΔG_solv_ |
| --- | --- | --- | --- | --- |
| Amoxicillin | -38.43 ± 5.35 | -311.87 ± 29.59 | -350.30 ± 32.74 | 316.55 ± 24.66 |
| [Silicristin](http://zinc12.docking.org/synonym/Silychristin) | -55.40 ± 3.44 | -32.14 ± 11.31 | -87.55 ± 10.47 | 37.01 ± 7.39 |
| Epigallocatechin 4-benzylthioether | -47.00 ± 6.23 | -38.94 ± 8.60 | -85.94 ± 12.55 | 40.12 ± 4.76 |
| Lysidicichin | -39.57 ± 4.41 | -29.01 ± 8.77 | -68.58 ± 9.95 | 35.04 ± 5.96 |
| [Epicatechin 3-O-(3-O-methylgallate)](http://zinc12.docking.org/synonym/Epicatechin%203-O-(3-O-methylgallate)) | -41.48 ± 5.60 | -34.51 ± 10.24 | -76.00 ± 10.84 | 40.93 ± 6.30 |
| [(-)-Gallocatechin gallate](http://zinc12.docking.org/synonym/(-)-Gallocatechin%20gallate) | -54.67 ± 5.03 | -41.24 ± 7.81 | -95.91 ± 9.19 | 51.69 ± 5.37 |

Table S2: Other energy components of the of top five phenolics following 120 ns MD simulation at the allosteric site of PBP2x of *S. pneumoniae*

Table S3: Average RMSD, ROG and SASA of top five phenolics following 120 ns MD simulation at the allosteric site of PBP2x of S*. pneumoniae*

| Systems | RMSD (Å) | ROG (Å) | SASA (Å) |
| --- | --- | --- | --- |
| Apo-PBP2x | 1.69 ± 0.15 | 24.17 ± 0.07 | 22380.62 ± 352.67 |
| Amoxicillin | 2.10 ± 0.21 | 24.31 ± 0.08 | 23064.71 ± 554.44 |
| [Silicristin](http://zinc12.docking.org/synonym/Silychristin) | 1.80 ± 0.15 | 24.16 ± 0.08 | 22783.07 ± 449.26 |
| Epigallocatechin 4-benzylthioether | 1.89 ± 0.19 | 24.08 ± 0.09 | 22256.15 ± 357.03 |
| Lysidicichin | 1.92 ± 0.22 | 24.28 ± 0.11 | 23298.86 ± 482.55 |
| [Epicatechin 3-O-(3-O-methylgallate)](http://zinc12.docking.org/synonym/Epicatechin%203-O-(3-O-methylgallate)) | 2.19 ± 0.23 | 24.13 ± 0.07 | 22749.56 ± 418.50 |
| [(-)-Gallocatechin gallate](http://zinc12.docking.org/synonym/(-)-Gallocatechin%20gallate) | 1.74 ± 0.15 | 24.21 ± 0.07 | 22928.39 ± 455.00 |

*
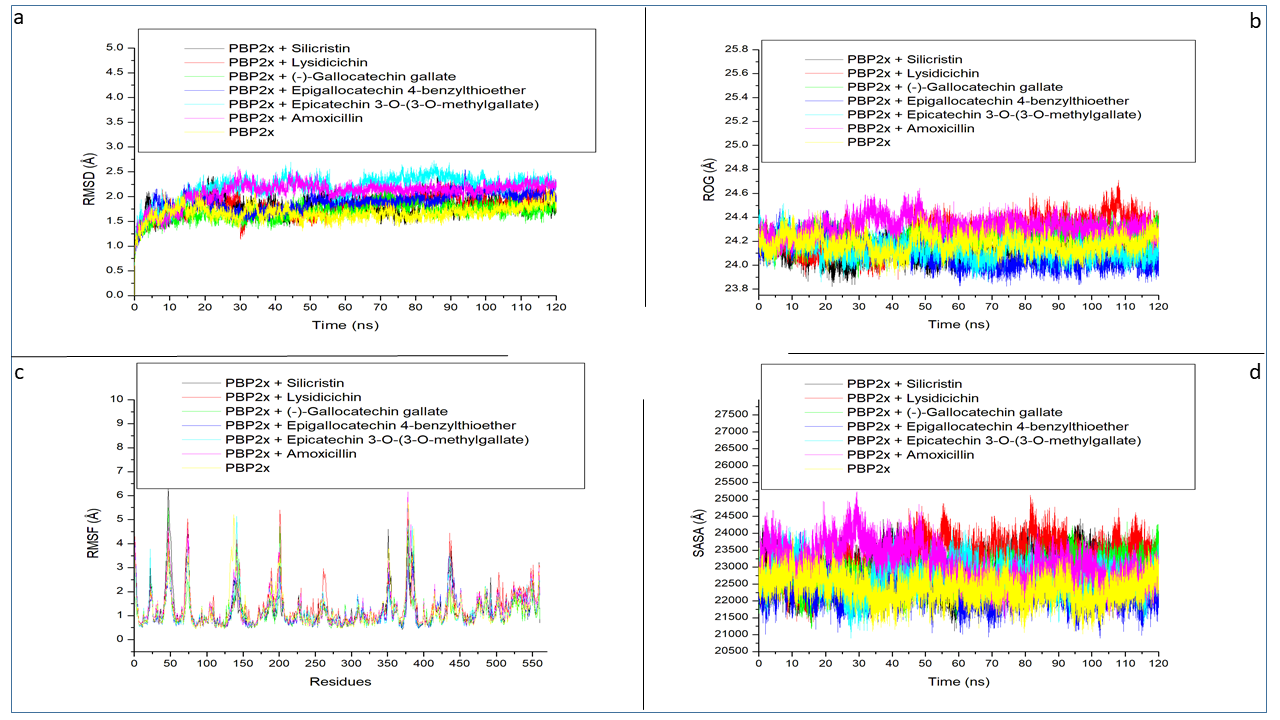
*

a

c

b

*

*

Figure S2: Comparative RMSD (a), ROG (b) and SASA (c) plot of top 5 lead phenolics against the allosteric site of PBP2x of *S. pneumoniae*


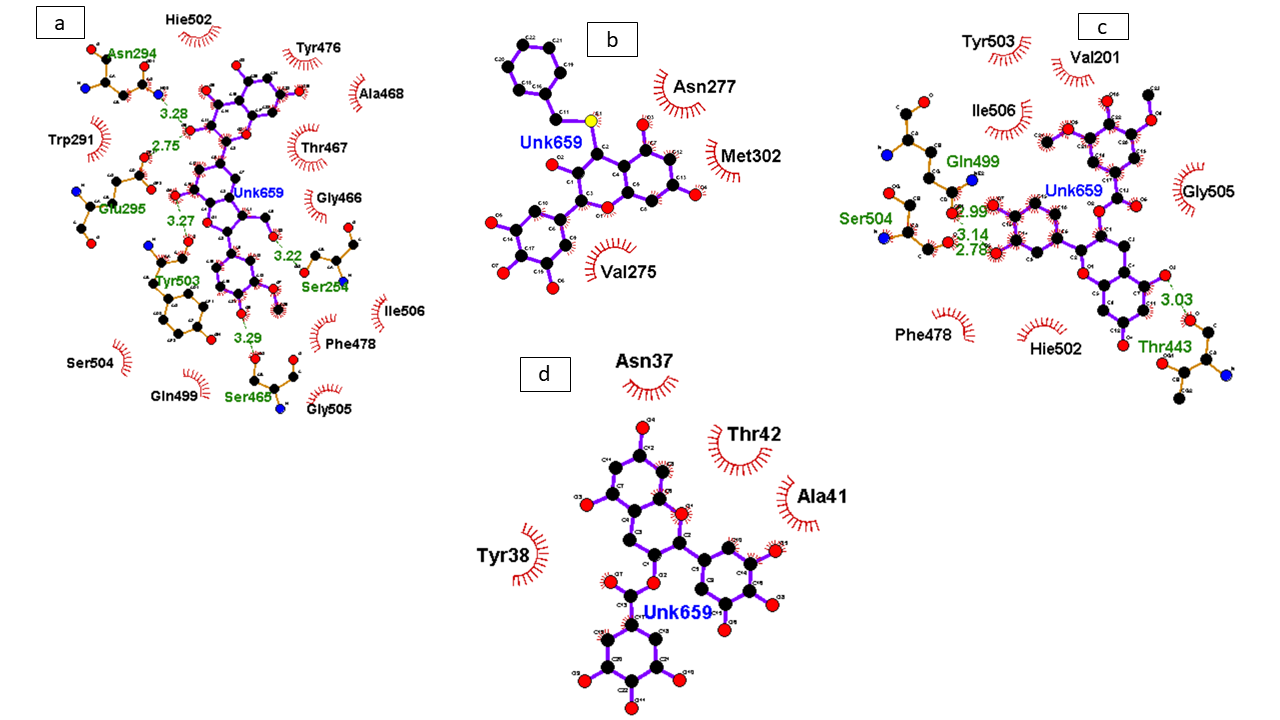


Figure S3: 2D plot of interactions of (a) [silicristin](http://zinc12.docking.org/synonym/Silychristin), (b) epigallocatechin 4-benzylthioether, (c) lysidicichin and (d) gallocatechin gallate at the active site of PBP2x after 120 MD simulation


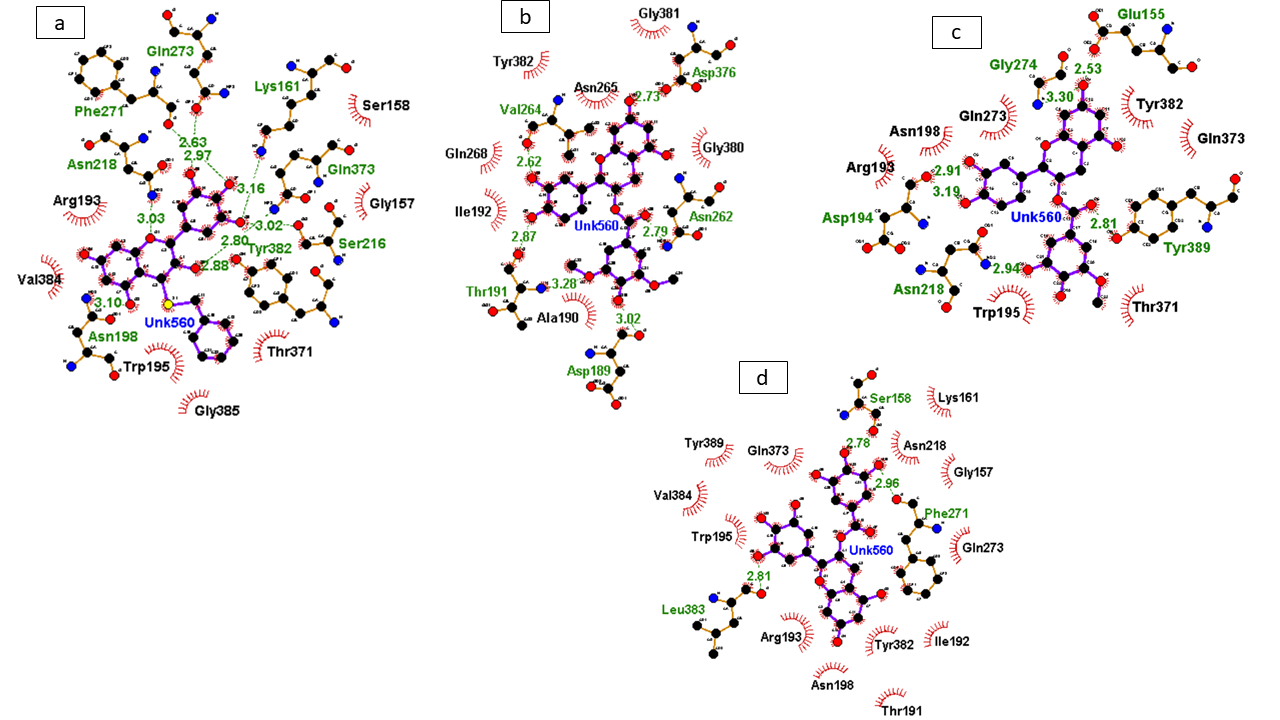


Figure S4: 2D plot of interactions of (a) epigallocatechin 4-benzylthioether, (b) lysidicichin, (c) [epicatechin 3-O-(3-O-methylgallate)](http://zinc12.docking.org/synonym/Epicatechin%203-O-(3-O-methylgallate)) and (d) gallocatechin gallate at the allosteric site of PBP2x after 120 MD simulation

Table S4: Interaction plots at different time intervals of the 120 ns MD simulation of best phenolics at active of PBP2x

| PBP2x + ECMG | |
| --- | --- |
| 0 ns | 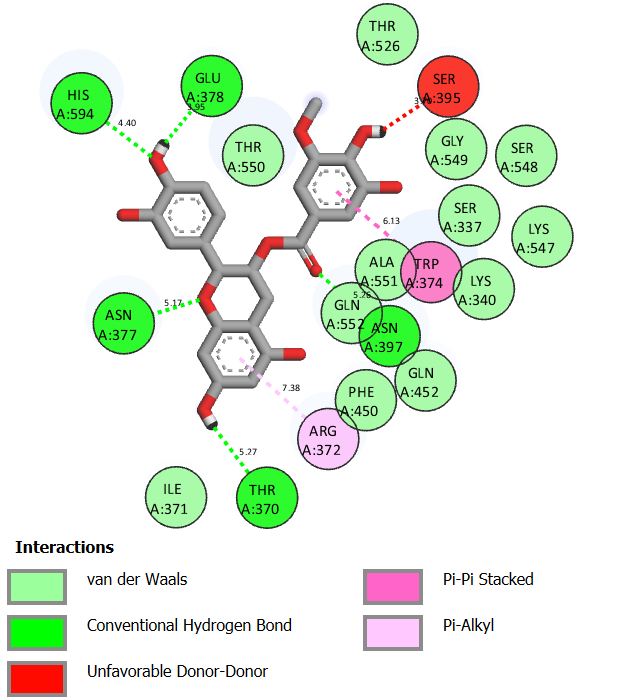 |
| 40 ns | 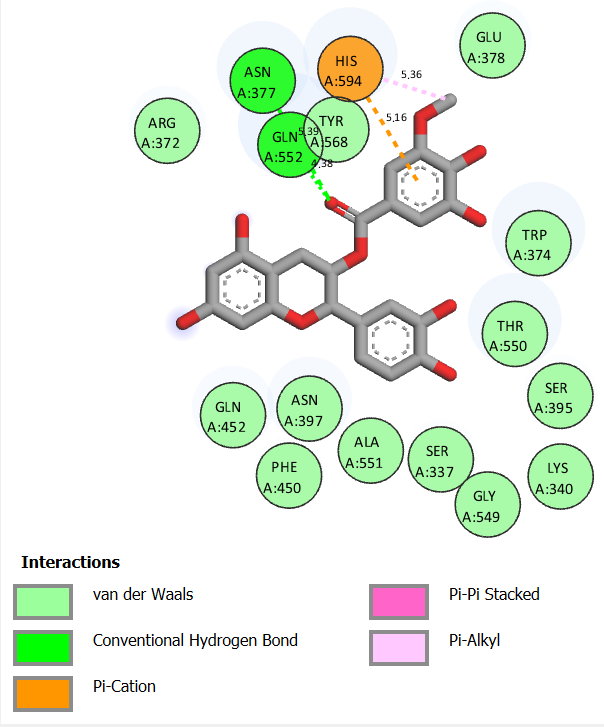 |
| 80 ns | 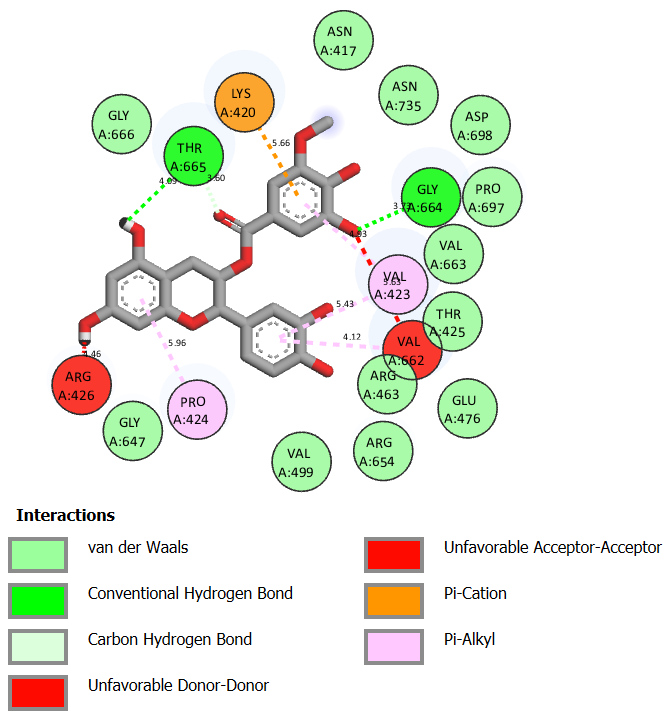 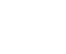 |
| 120 ns | 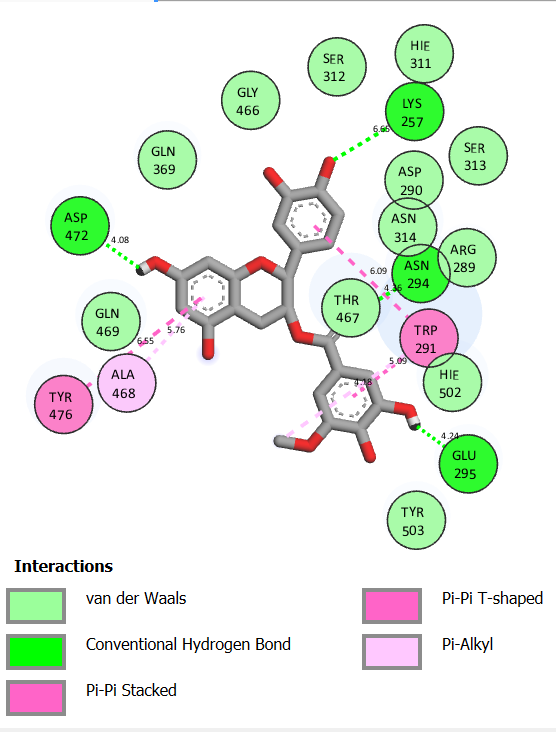 |
| PBP2x + Amoxicillin | |
| 0 ns | 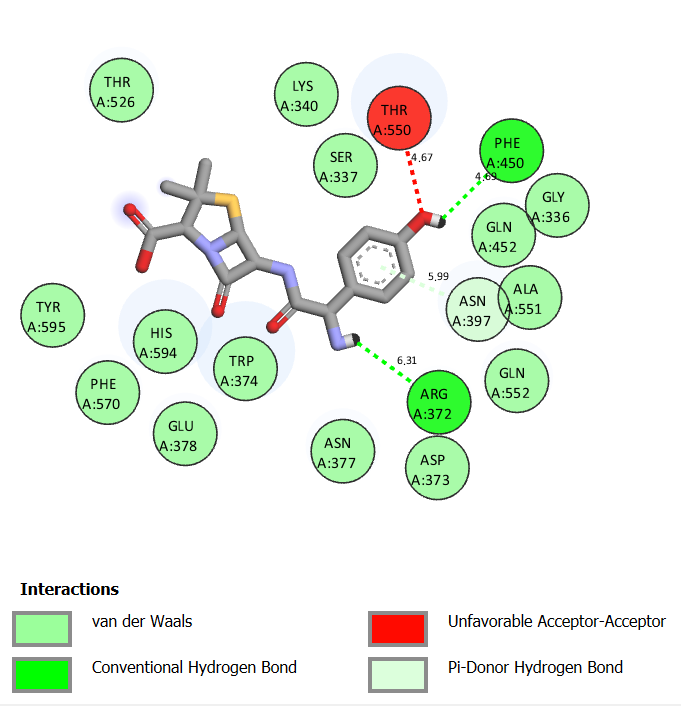 |
| 40 ns | 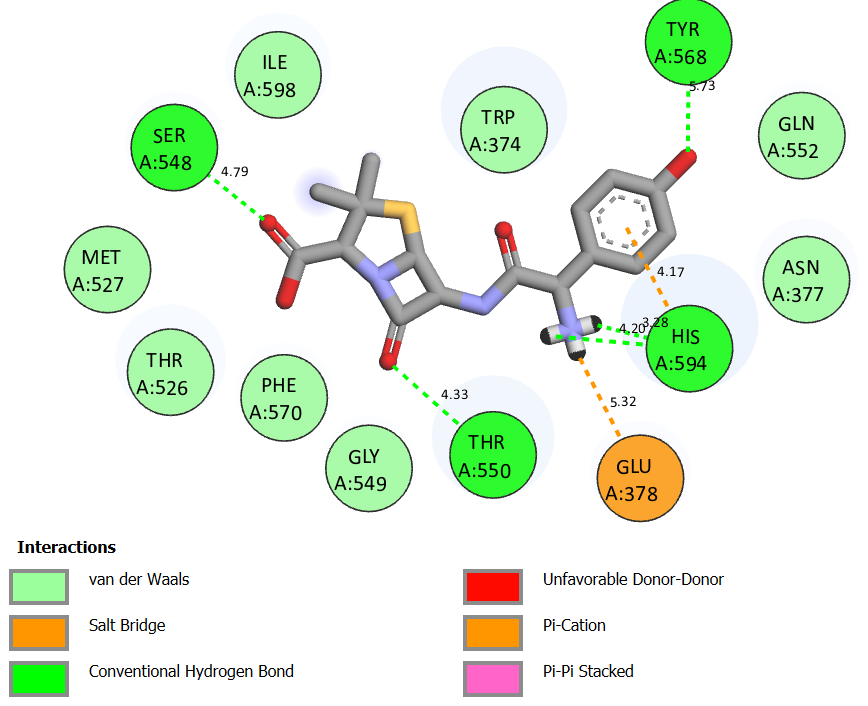 |
| 80 ns | 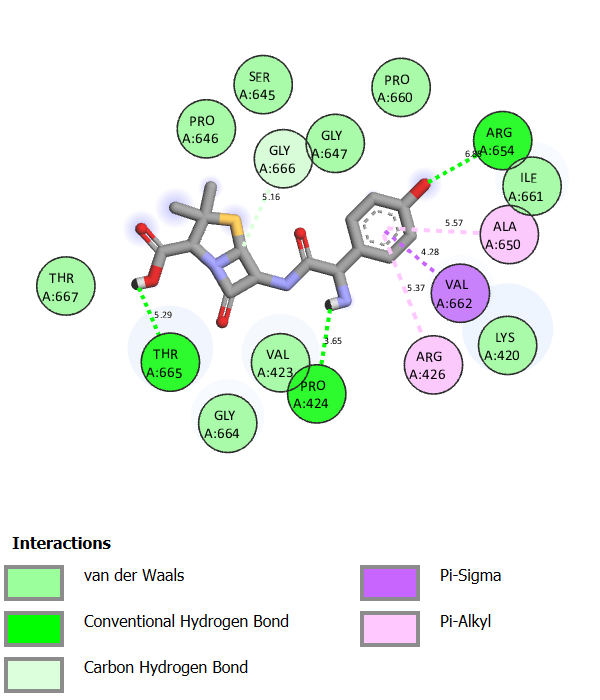 |
| 120 ns | 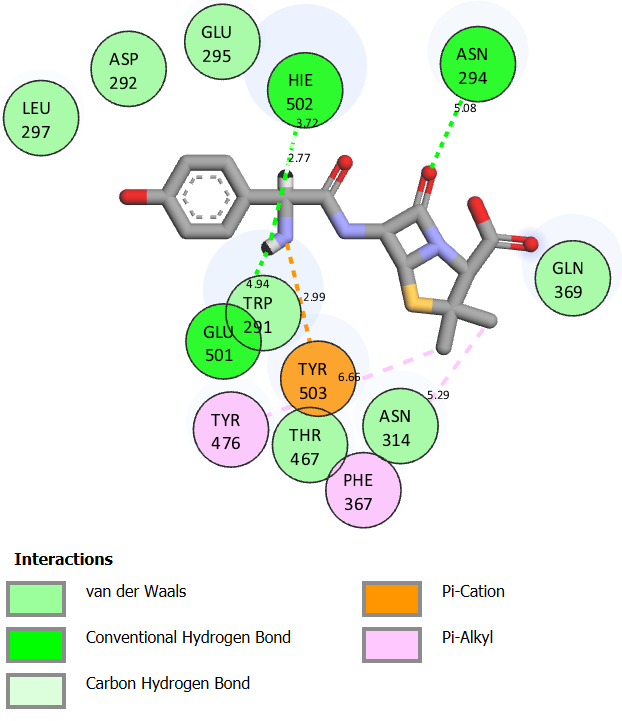 |
